# Management of Infants Exposed to Prenatal and Postnatal Elexacaftor/Tezacaftor/Ivacaftor: A Cross‐Sectional Survey

**DOI:** 10.1002/hsr2.72332

**Published:** 2026-04-27

**Authors:** Haley Haskett, Clement L. Ren

**Affiliations:** ^1^ Division of Pulmonary and Sleep Medicine, Children's Hospital of Philadelphia Philadelphia Pennsylvania USA

## 1 Introduction

Prenatal and postnatal exposure to elexacaftor/tezacaftor/ivacaftor (ETI) is an emerging and increasingly important clinical issue in infants with and without cystic fibrosis (CF). The number of pregnant women with CF taking ETI has markedly increased, leading to a concomitant increase in infants exposed to ETI in utero [1]. Prenatal ETI therapy in women who are pregnant with a fetus prenatally diagnosed with CF is also leading to infants with in utero ETI exposure [2, 3, 4, 5, 6]. In both cases, infants can continue to be exposed to ETI postnatally if their mothers are taking ETI while breastfeeding.

Although a growing number of reports describe prenatal ETI exposure and early outcomes [2, 3, 4, 5, 6, 7, 8, 9, 10, 11, 12, 13, 14, 15], these reports do not capture the broader landscape of clinical practice across CF centers. Currently, there are no published guidelines or recommendations for the management of these infants, leaving a gap in understanding how clinicians approach postnatal care for ETI‐exposed infants. As this population grows, the absence of standardized practices presents challenges for both clinicians and families navigating an evolving and uncertain clinical territory. An increased understanding of how CF centers are managing infants exposed to prenatal and/or postnatal ETI would help inform clinicians caring for these infants about the current state of practice.

The objectives of this study were to characterize the experience of CF centers in the United States in caring for ETI‐exposed infants, describe current postnatal management practices, and assess the need for clinical resources and guidance. Through a national survey of CF centers, we aimed to better understand how centers are navigating this emerging clinical problem, identify common practices and gaps, and inform future efforts to develop evidence‐based recommendations for the care of ETI‐exposed infants.

## 2 Methods

An online survey was distributed through the US CF Foundation (CFF) to CF care team members at CFF‐accredited care centers in 2024. The survey link was emailed to program directors and coordinators at each center and shared with relevant team members. More than one response could be submitted from a given center. One follow‐up reminder email was sent 2 weeks after the initial distribution, and all responses were collected over a 4‐week period. The survey collected the respondent's role, state, and affiliated CF center, as well as their experience and approach to the postnatal management of infants with prenatal and/or postnatal ETI exposure. CF care center type was categorized according to standard CFF classifications as adult, pediatric, or affiliate. Affiliate centers were defined as regional programs associated with a primary CFF‐accredited center, sharing oversight and resources while providing local care [16]. Data were collected and managed using REDCap electronic database. Descriptive statistics were used to analyze the survey responses. Categorical variables are presented as counts and percentages, calculated using the number of respondents to each question as the denominator; missing responses were excluded from percentage calculations. Percentages were rounded to the nearest whole number. The study was deemed exempt from Institutional Review Board oversight (IRB 24‐022849). Participation was voluntary, and completion of the anonymous online survey implied informed consent.

## 3 Results

Thirty‐three (12%) of 287 centers responded (7 adult, 19 pediatric, and 7 affiliate). Thirty‐two respondents represented different centers, while one respondent did not identify an affiliated center. The respondents represented a variety of roles and geographic regions (Table 1). The majority of respondents (76%) reported caring for an infant with ETI exposure or a mother with CF taking ETI while breastfeeding. Thirty percent had cared for infants who received fetal therapy. Most respondents (55%) indicated that their state newborn screening (NBS) program does not have a process in place for follow‐up of infants exposed to in utero ETI, and 27% reported their CF center lacks a follow‐up protocol. There was variability in reported responsibility for counseling and follow‐up testing. Both adult (58%) and pediatric (42%) CF centers were responsible for counseling, while pediatric CF centers (61%) were most often responsible for ordering follow‐up testing. Common recommended testing included genetic testing, sweat chloride testing, liver function tests, and eye exams (Table 2). In free‐text responses, respondents emphasized the need for genetic testing, structured follow‐up, parental education, and standardized guidelines. When asked what resources would be most helpful, over half suggested standardized guidelines or consensus statements, followed by educational materials for providers and families, and additional published research (Table 3).

**Table 1:** Characteristics of respondents.

Role	
Physician	20 (61%)
Advanced practice provider	3 (9%)
Nurse	8 (24%)
Pharmacist	1 (3%)
Dietician	1 (3%)
CF center patient population	
Adult	7 (21%)
Pediatric	19 (58%)
Both	7 (21%)
Region	
Northeast	5 (15%)
Southeast	8 (24%)
Midwest	11 (33%)
Southwest	2 (6%)
West	6 (18%)
Unknown	1 (3%)

Abbreviation: CF, cystic fibrosis.

**Table 2:** Multiple‐choice responses.

Have you been involved in the care/evaluation of an infant exposed to in utero ETI?	
Yes	22 (67%)
No	11 (33%)
What reason was the infant exposed to in utero ETI?	
Infant born to a mother with CF	12 (36%)
Infant who received fetal therapy	4 (12%)
Both	6 (18%)
Does your state NBS program have a process in place for infants exposed to in utero ETI?	
Yes	8 (24%)
No	18 (55%)
Unsure	7 (21%)
Does your CF center have a process in place for follow‐up of infants exposed to in utero ETI?	
Yes	23 (70%)
No	9 (27%)
Unsure	1 (3%)
Who is primarily responsible to counsel the mom about follow‐up testing if her baby has been exposed to in utero and/or breast milk ETI?	
Adult CF center	19 (58%)
Pediatric CF center	14 (42%)
PCP	0 (0%)
Who is responsible for ordering any follow‐up testing for infants exposed to in utero and/or breast milk ETI?	
Adult CF center	5 (15%)
Pediatric CF center	20 (61%)
PCP	8 (24%)
Which of the following tests do you recommend for infants exposed to in utero ETI?	
Genetic testing	27 (82%)
Sweat test	22 (67%)
Fecal elastase	12 (36%)
Liver function tests	23 (70%)
Lipase/amylase	8 (24%)
Eye exam	26 (79%)
ETI levels	1 (3%)
Have you taken care of any lactating moms with CF on ETI and/or infants exposed to breast milk ETI?	
Yes	20 (61%)
No	11 (33%)
Unsure	2 (6%)
Which of the following tests do you recommend for infants exposed to breast milk ETI?	
Sweat test	6 (18%)
Fecal elastase	3 (9%)
Liver function tests	12 (36%)
Lipase/amylase	2 (6%)
Eye exam	14 (42%)
ETI levels	0 (0%)
None	2 (6%)
Have you been involved in a case where an infant had adverse effects attributable to ETI?	
Yes	3 (9%)
No	27 (82%)
Unsure	3 (9%)
If so, what types of adverse effects have you seen in infants related to ETI?	
Abnormal liver function tests	3 (9%)
Abnormal eye exam	1 (3%)

Abbreviations: CF, cystic fibrosis; ETI, elexacaftor/tezacaftor/ivacaftor; NBS, newborn screening; PCP,

primary care provider.

**Table 3:** Representative free‐text responses.

Do you have any comments about evaluation of infants exposed to in utero and/or breast milk ETI?	
Genetic testing is necessary for ETI‐exposed infants	5 (15%)
Structured follow‐up of these infants is needed	4 (12%)
Standardized guidelines for monitoring are needed	4 (12%)
Parental education is important	2 (6%)
Education needs to be provided to PCP	2 (6%)
What resources would be helpful to providers facing these scenarios?	
Standardized guidelines/consensus statements	18 (55%)
Handouts for parents and PCPs	8 (24%)
Published data	3 (11%)

Abbreviations: ETI, elexacaftor/tezacaftor/ivacaftor; PCP, primary care provider.

## 4 Discussion

This study shows that many CF centers have encountered infants with prenatal and/or postnatal ETI exposure and highlights a wide range of practices regarding their care. While most respondents reported experience with ETI‐exposed infants, there was considerable variability in postnatal management. Furthermore, many CF centers and state NBS programs lacked standardized protocols for these infants. Although multiple papers describe outcomes of fetal therapy and in utero ETI exposure [2, 3, 4, 5, 6, 7, 8, 9, 10, 11, 12, 13, 14, 15], this survey represents the first to characterize how US. CF centers are approaching the evaluation and management of these infants in clinical practice.

Responsibility for counseling and follow‐up testing varied across centers. Adult CF centers were more commonly responsible for parental counseling, while pediatric CF centers were more commonly responsible for ordering testing, with some involvement from primary care providers. This division likely reflects differences in institutional structure but also underscores the need for role delineation and inter‐provider collaboration.

Case reports have described adverse effects in ETI‐exposed infants, including liver enzyme abnormalities and cataracts [11, 12, 14]. In our survey, three centers reported observing adverse effects, specifically abnormal liver function tests and one case of an abnormal eye exam. Despite concerns about adverse effects, standardized guidelines for follow‐up of ETI‐exposed infants do not currently exist. Few reports have recommended monitoring, particularly liver function tests and ophthalmologic status [1, 3, 11, 12, 14, 17, 18, 19]. In our survey, commonly recommended testing included genetic testing, sweat chloride testing, liver function tests, and eye exams. However, variability in the recommendation of these tests underscores the lack of consensus in postnatal management. In addition to adverse events reported in humans, animal data suggest that in utero ETI exposure can affect gene expression in the developing fetal brain [20]. The significance of these findings in humans is unclear, but this is another aspect of potential adverse effects that needs further study.

Free‐text responses underscored key areas of need and concern among providers caring for ETI‐exposed infants. Many respondents expressed the importance of genetic testing and structured follow‐up for these infants. The need for enhanced parental education was also emphasized, particularly regarding the potential risks associated with ETI exposure and the importance of postnatal monitoring. Notably, there was a strong call for the development of standardized guidelines and consensus statements to promote consistent, evidence‐based care. Collectively, these insights point to a critical opportunity for professional societies and researchers to provide recommendations and educational resources to support providers navigating this evolving clinical territory. Data from studies of infants exposed to in utero ETI will also help guide management in the future [4, 21].

Limitations of this study include a low response rate, which may limit generalizability and introduce selection bias. Centers with more experience or concern regarding ETI‐exposed infants may have been more likely to respond, further contributing to potential bias. Additionally, all data were self‐reported, and we did not independently verify clinical practices or collect objective data. Despite these limitations, our results highlight the frequency of this clinical problem, the variation in care, and the need for additional guidance and evidence for the care of these infants.

Several reports have highlighted the potential benefits of fetal therapy. Case reports and small case series suggest that in utero ETI exposure can mitigate early manifestations of CF, including meconium ileus, and preserve organ function such as exocrine pancreatic function and development of the vas deferens [22, 23, 24]. These findings raise the possibility that prenatal ETI therapy could modify early disease pathophysiology and improve long‐term outcomes. However, these potential benefits must be weighed against limited safety data. Animal studies suggest that in utero ETI exposure may influence gene expression in the developing fetal brain, although the clinical significance of these findings in humans remains unclear [20]. Given the potential for both benefit and risk, it is essential that the CF community develop consistent approaches to counseling, monitoring, and follow‐up of ETI‐exposed infants. Standardized practices will be important to ensure equitable access to emerging therapies and to allow systematic tracking of maternal treatment, prenatal ETI exposure, and clinical outcomes across centers in the US and internationally.

In summary, our survey results show that management of infants with in utero ETI exposure occurs frequently at US CF care centers and that there is substantial variability in monitoring and management of these infants. There is a clear need for standardized guidelines, improved care coordination, and greater educational support for both healthcare professionals and families. As ETI use during pregnancy and breastfeeding becomes increasingly common, the CF community must work collaboratively to define standardized practices for evaluation and follow‐up of ETI‐exposed infants.

## Author Contributions

**Haley Haskett:** conceptualization, investigation, writing – original draft, writing – review and editing, funding acquisition. **Clement L. Ren:** conceptualization, writing – original draft, writing – review and editing, supervision, funding acquisition.

## Conflicts of Interest

The authors declare no conflicts of interest.

## Transparency Statement

The corresponding author, Haley Haskett, affirms that this manuscript is an honest, accurate, and transparent account of the study being reported; that no important aspects of the study have been omitted; and that any discrepancies from the study as planned (and, if relevant, registered) have been explained.

## Supporting information


**Supporting File:** hsr272332‐sup‐0001‐supmat.docx.

## Data Availability

The data that support the findings of this study are available from the corresponding author upon reasonable request.
