# Supplementary material for: Management of Infants Exposed to Prenatal and Postnatal Elexacaftor/Tezacaftor/Ivacaftor: A Cross‐Sectional Survey
Source: Health Sci Rep. 2026 Apr 27;9(5):e72332. doi: 10.1002/hsr2.72332 (PMC13121855; doi:10.1002/hsr2.72332)
Supplement: Supplementary file 1 — Supporting File: hsr272332‐sup‐0001‐supmat.docx. [file HSR2-9-e72332-s001.docx]

Supplementary Table 1. Survey Questions

| 1. Please select your state | | |
| --- | --- | --- |
| 2. Please select your CF program | | |
| 3. Role | | |
|  | Physician |  |
|  | Advanced practice provider |  |
|  | Nurse |  |
|  | Pharmacist |  |
|  | Other |  |
| 4. I take care of | | |
|  | Pediatric PwCF only |  |
|  | Adult PwCF only |  |
|  | Both pediatric and adult PwCF |  |
| 5. Have you been involved in the care or evaluation of an infant exposed to in utero ETI? | | |
|  | Yes |  |
|  | No |  |
|  | Unsure |  |
| 6. What reason was the infant exposed to in utero ETI? Check all that apply | | |
|  | Infant born with mother with CF |  |
|  | Infant who received fetal therapy |  |
|  | N/A |  |
| 7. Does your state’s NBS program have a process in place for infants exposed to in utero ETI? | | |
|  | Yes |  |
|  | No |  |
|  | Unsure |  |
| 8. Does your CF Center have a process in place for follow up of infants exposed to in utero ETI? | | |
|  | Yes |  |
|  | No |  |
|  | Unsure |  |
| 9. Who is primarily responsible to counsel the mom about follow up testing if her baby has been exposed to in utero and/or breast milk ETI? | | |
|  | Adult CF center |  |
|  | Pediatric CF center |  |
|  | PCP |  |
| 10. Who is responsible for ordering any follow up testing for infants exposed to in utero and/or breast milk ETI? | | |
|  | Adult CF center |  |
|  | Pediatric CF center |  |
|  | PCP |  |
| 11. How are you notified of infants exposed to in utero ETI? | | |
|  | Adult CF center |  |
|  | Pediatric CF center |  |
|  | PCP |  |
| 12. Which of the following tests do you recommend for infants exposed to in utero ETI? Check all that apply | | |
|  | Genetic testing |  |
|  | Sweat test |  |
|  | Fecal elastase |  |
|  | Liver function tests |  |
|  | Lipase/amylase |  |
|  | Eye exam |  |
|  | ETI levels |  |
|  | Other |  |
|  | None |  |
| 13. Have you taken care of any lactating moms with CF on ETI and/or infants exposed to breast milk ETI? | | |
|  | Yes |  |
|  | No |  |
|  | Unsure |  |
| 14. Which of the following tests do you recommend for infants exposed to breast milk ETI? Check all that apply | | |
|  | Sweat test |  |
|  | Fecal elastase |  |
|  | Liver function tests |  |
|  | Lipase/amylase |  |
|  | Eye exam |  |
|  | ETI levels |  |
|  | Other |  |
|  | None |  |
| 15. Do you have any thoughts and/or comments about evaluation of infants exposed to in utero or breast milk ETI? | | |
| 16. Have you been involved in a case where an infant had adverse effects attributable to ETI? | | |
|  | Yes |  |
|  | No |  |
|  | Unsure |  |
|  | N/A |  |
| 17. Please check all that apply | | |
|  | Abnormal liver function tests |  |
|  | Abnormal eye exam |  |
|  | Other |  |
| 18. What resources would be helpful to providers facing these scenarios? | | |

Abbreviations used: CF, cystic fibrosis; PwCF, people with cystic fibrosis; ETI,

elexacaftor/tezacaftor/ivacaftor; NBS, newborn screening; PCP, primary care provider
